# Association between serum vitamin D and severe headache or migraine: A population-based analysis

**DOI:** 10.1371/journal.pone.0313082

**Published:** 2025-01-03

**Authors:** Shunfa Hao, Renyi Qian, Yiru Chen, Jingfang Liu, Xiaoyan Xu, Yunxiang Guan

**Affiliations:** 1 Department of Encephalopathy, The First Affiliated Hospital of Henan University of Traditional Chinese Medicine, Zhengzhou, China; 2 The First Clinical Medical School, Henan University of Traditional Chinese Medicine, Zhengzhou, China; Universidade de Sao Paulo Faculdade de Saude Publica, BRAZIL

## Abstract

**Background:**

Vitamin D is thought to play a role in the development of migraine, but the nature of the relationship is still not fully understood. Although some studies have shown an association between vitamin D deficiency and migraine, other studies have had inconsistent or inconclusive results. Therefore, further research is needed to better understand the relationship between vitamin D and migraine headaches.

**Methods:**

We selected 9142 participants from the 2001–2004 National Health and Nutrition Examination Survey (NHANES). In our study, the term "serum vitamin D" refers to the concentration of 25OHD2 + 25OHD3 (nmol/L) in the blood. Migraine was assessed based on self-reports in the miscellaneous pain section of the NHANES questionnaire. Associations between vitamin D and the risk of migraine were examined using multiple logistic regression, smoothed curve fitting, and stratified analyses.

**Results:**

In our study, 20.53% of the participants suffered from migraine. The prevalence of migraine was higher in those with lower serum vitamin D levels. Participants in the highest quartile of serum vitamin D levels were found to have a 16% lower prevalence than those in the lowest quartile in the fully adjusted model (OR = 0.84, 95% CI 0.71–0.99). This result was supported by stratified analysis and smoothed curve fitting.

**Conclusion:**

Our study showed a significant negative correlation between serum vitamin D levels and the prevalence of migraine in American adults.

## Background

A common primary headache, migraine is characterized by episodes of moderate to severe headache that persist for 4 to 72 h. The headache is usually pounding and unilateral, and it is frequently accompanied by nausea, photophobia, and/or phonophobia. According to a review, 2.5% of episodic migraine attacks develop into chronic migraine, and the prevalence of migraine is 14.4% worldwide [1]. Migraine affects more than a billion people worldwide, with the highest prevalence in Southeast Asia (25%-35%) and the lowest in China (9%) [2]. Currently, evidence supports that abnormal releases of several neurotransmitters, such as glutamate, 5-hydroxytryptamine, and vasoactive peptides, from the nervous system cause migraine attacks. These releases can cause vasodilatation, abnormal neuronal excitation, and increased inflammatory responses [3,4]. A growing body of research in the last several years has demonstrated a substantial correlation between dietary consumption and the frequency and beginning of migraine attacks [5–8]. For example, diets high in salt and low in vitamins and minerals (e.g., magnesium) may increase the risk of migraine.

Vitamin D is a fat-soluble vitamin whose main sources include sunlight exposure, food intake, and supplements [9]. By attaching to the vitamin D receptor, which controls the body’s intake and metabolism of calcium and phosphorus, vitamin D preserves bone health [10]. Furthermore, an increasing amount of evidence indicates that vitamin D may potentially affect how the neurological system functions, such as controlling neuronal activity, reducing inflammatory reactions, and altering vascular function [11,12]. As a result, vitamin D is crucial for maintaining bone health and may influence the development of migraines.

Supplementing with vitamin D may help lessen the frequency and intensity of migraine attacks, according to many clinical investigations [13–15]. Consequently, the possible correlation between blood vitamin D levels and migraine occurrence is evaluated in this paper using data from the NHANES.

## Methods

### Study population

**Fig 1:**
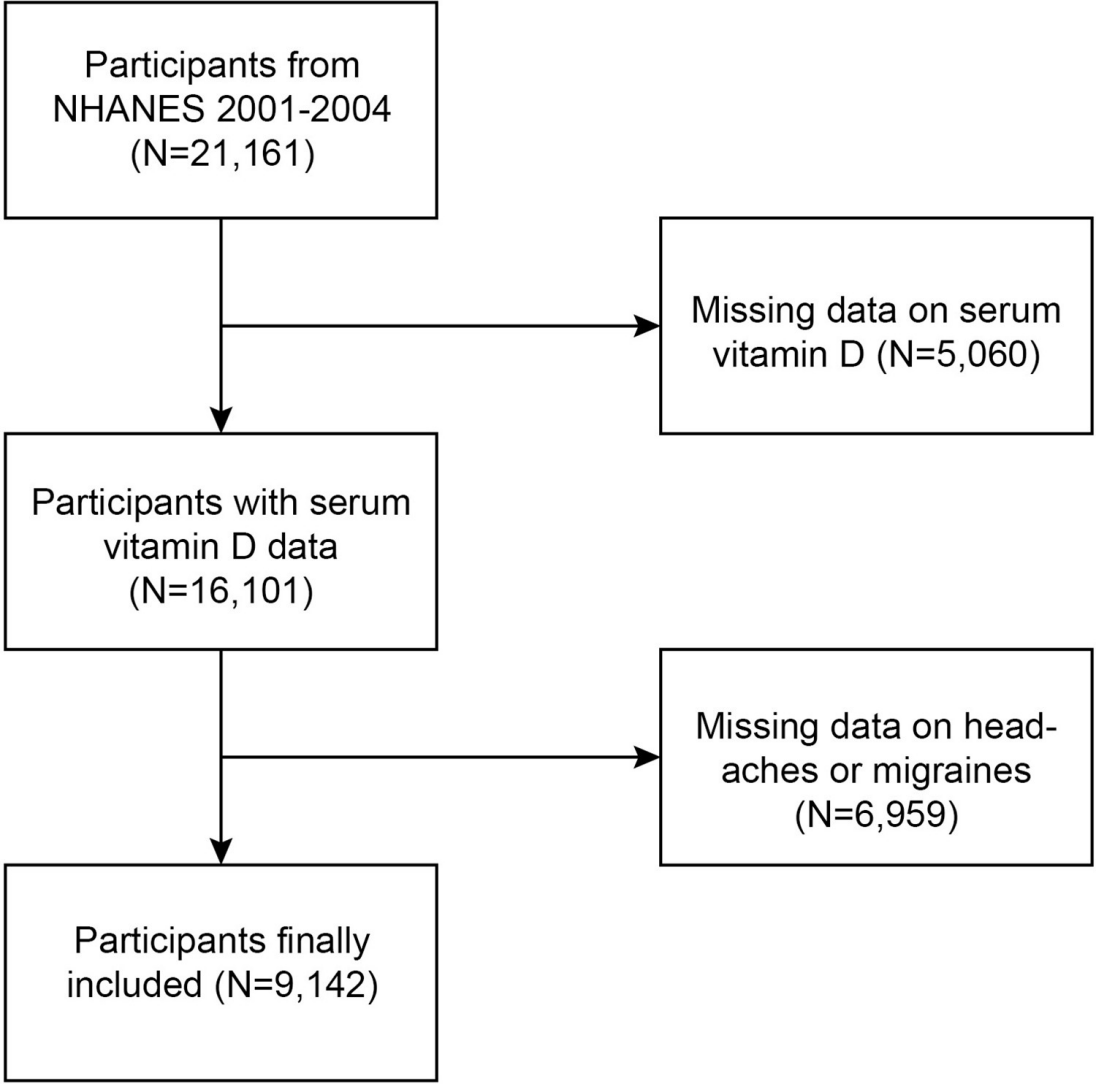
The flow chart of the selection of included studies.

NHANES is a cross-sectional, stratified, multistage probability survey conducted by the National Center for Health Statistics (NCHS) of the U.S. Centers for Disease Control and Prevention (CDC). The survey protocol was approved by the NCHS Institutional Review Board, and all participants provided written informed consent.21,161 participants were recruited for NHANES in 2001–2004. After excluding those with missing migraine data (10,713) and those with missing serum vitamin D data (1,571), 9,142 were eligible for our final analysis (Fig 1).

### Outcome variable

Migraine assessment: Severe headache or migraine is based on self-report from the Miscellaneous Pain section of the NHANES questionnaire. Participants who answered "yes" to the question "In the past 3 months, have you had severe headaches or migraines?" were categorized as having severe headaches or migraines. Given that most individuals with severe headaches or migraines are diagnosed with migraines, it is reasonable to classify these respondents as migraineurs [16,17].

### Measurement of serum vitamin D concentration

Utilizing ultra-high-performance liquid chromatography-tandem mass spectrometry (UHPLC-MS/MS), usually with one of the three pentafluorophenyl (PFP) columns between subjects, the assay is based on the quantitative determination of 25-hydroxyvitamin D3 (25OHD3), epi-25-hydroxyvitamin D3 (epi-25OHD3), and 25-hydroxyvitamin D2 (25OHD2) in human serum. Chromatography was used to separate the analytes amongst individuals on one of the three pentafluorophenyl (PFP) columns. The three columns’ mobile phase compositions differed significantly, but the water’s methanol level ranged from 69% to 72%. The mobile phase’s composition must be the same for the solution added to the serum before extraction, the solution used for reconstitution, and the needle wash. This study defined "serum vitamin D" as the blood level of 25OHD2+25OHD3 measured in nanograms per milliliter.

### Measurements of covariates

Our analysis included other confounders known to potentially affect the association between serum vitamin D and migraine. The covariates included gender, age, race (Mexican American, other Hispanic, non-Hispanic white, non-Hispanic black, or other race), education level (not in high school, high school, or >high school), poverty-to-income ratio(PIR), body mass index(BMI), smoking status (defined as daily, occasionally, or never), and alcohol consumption (defined as 12 drinks or more annually), hypertension status, hyperlipidemia status, diabetes status, and stroke status. A person’s BMI can be computed by dividing their height in meters squared by their weight in kilograms. Self-reported physician diagnosis (yes/no) was used to determine the presence of hypertension, hyperlipidemia, diabetes mellitus, and stroke.

### Statistical analysis

Categorical variables are often given as numbers (percentages), and continuous variables as means (standard deviation, SD). For continuous variables, we performed the Kruskal-Wallis rank sum test, while for count variables with theoretical counts less than 10, Fisher’s exact probability test was used. P values less than 0.05 were regarded as statistically significant for statistical analysis. Multivariate logistic regression models were built in this study to further analyze the relationship between serum vitamin D levels and migraine: model 1 was unadjusted; model 2 was adjusted for gender, age, and race; model 3 was adjusted for gender, age, race, education level, BMI, PIR, hypertension, and hyperlipidemia, diabetes, stroke, alcohol use, and Smoking Behavior. In addition, subgroup analyses and smoothed curve fitting were performed according to gender, age, PIR, ethnicity, body mass index, and hypertension status to further investigate variables affecting the relationship between serum vitamin D and migraine. R version 3.4.3 (http://www.R-project.org, The R Foundation) and EmpowerStats software (http://www.empowerstats.com; X&Y Solutions, Inc., Boston, MA) were used for all analyses.

## Results

### Baseline characteristics

In all, 9142 individuals were co-enrolled in the research. The clinical features of the patients are listed in Table 1, along with a column that displays the stratified groups according to serum vitamin D quartiles. Serum vitamin D quartiles 1–4 ranges were 9.1–40.9, 40.9–55.7, 55.7–70.6, and 70.6–198.0. The top quartile had greater PIR(2.96 ± 1.60), a higher number of Mexican Americans(78.46%), a higher percentage of high school graduates(27.43%) and highly educated individuals(51.54%), Higher proportion of male(46.29%), higher proportion of drinkers(74.23%) as compared to the bottom quartile. On the other hand, their BMI(26.65 ± 5.09) was lower, their prevalence of hypertension(28.07%), hyperlipidemia(36.09%), diabetes(7.25%), and stroke(3.28%) was lower, and their percentage of smokers was lower. All differences between quartiles were statistically significant with P-values less than 0.05.

**Table 1:** Baseline characteristics of participants in NHANES 2001–2004 (n = 9142).

Participants (n)	serum vitamin D				*P* value
	Q1(9.1–40.9)	Q2(40.9–55.7)	Q3(55.7–70.6)	Q4(70.6–198)	
Gender, n (%)					<0.001
Male	942(42.00)	1096(50.53)	1264(52.98)	1085(46.29)	
Female	1301(58.00)	1073(49.47)	1122(47.02)	1259(53.71)	
Age, Mean ± SD	48.96 ± 18.84	50.28 ± 19.38	51.09 ± 19.19	48.79 ± 19.32	<0.001
Race/ethnicity, n (%)					<0.001
Non-Hispanic White	523 (23.32)	551 (25.40)	529 (22.17)	288 (12.29)	
Non-Hispanic Black	76 (3.39)	85 (3.92)	109 (4.57)	64 (2.73)	
Mexican American	561 (25.01)	1019(46.98)	1459(61.15)	1839(78.46)	
Other Hispanic	977 (43.56)	404 (18.63)	216 (9.05)	96 (4.10)	
Other/Mixed	106 (4.73)	110 (5.07)	73 (3.06)	57 (2.43)	
Education level, n (%)					<0.001
<High school	834 (37.18)	693 (31.95)	353 (14.79)	220 (9.39)	
High school	519 (23.14)	512 (23.61)	547 (22.93)	643 (27.43)	
>High school	890 (39.68)	964 (44.44)	1158(48.53)	1208(51.54)	
PIR, Mean ± SD	2.21 ± 1.50	2.53 ± 1.60	2.75 ± 1.63	2.96 ± 1.60	<0.001
BMI, Mean ± SD	30.10 ± 7.49	28.77 ± 6.03	27.75 ± 5.41	26.65 ± 5.09	<0.001
Hypertension, n (%)	797 (35.53)	734 (33.84)	735 (30.80)	658 (28.07)	<0.001
Hyperlipidemia, n (%)	838 (37.36)	816 (37.62)	965 (40.44)	846 (36.09)	<0.001
Diabetes, n (%)	340 (15.16)	291 (13.42)	240 (10.06)	170 (7.25)	<0.001
Stroke, n (%)	102 (4.55)	71 (3.27)	71 (2.98)	77 (3.28)	0.020
Drinking status, n (%)	1401(62.46)	1437(66.25)	1677(70.28)	1740(74.23)	<0.001
Smoke status, n (%)					<0.001
Every day	978 (43.60)	796 (36.70)	762 (31.94)	789 (33.66)	
Some days	210 (9.36)	201 (9.27)	222 (9.30)	213 (9.09)	
Not at all	1055(47.04)	1172(54.03)	1402(58.76)	1342(57.25)	
Migraine, n (%)	524 (23.36)	466 (21.48)	440 (18.44)	447 (19.07)	<0.001

Continuous variable: Mean ± standard deviation; serum vitamin D was classified according to quartiles, Q1 to Q4 (Q1 ≤40.90nmol/L; 40.90nmol/L < Q2 ≤ 55.70 nmol/L;55.70 nmol/L < Q3 ≤ 70.60 nmol/L; Q4 > 70.6 nmol/L). p-value: For continuous variables, Kruskal Wallis rank sum tests were performed and Fisher’s exact probability tests were performed for count variables with theoretical counts less than 10.

Q, quartile; PIR, ratio of family income to poverty; BMI, body mass index.

### Relationship between serum vitamin D levels and migraine

Table 2 shows the relationship between serum vitamin D levels and migraine. Participants were grouped according to serum vitamin D quartiles and analyzed using Q 1 as the reference group. In Model 1, no adjustment was made for any of the variables. Serum vitamin D was significantly associated with migraine in Q 4 compared to Q 1, with an OR and 95% CI of 0.77 (0.67, 0.89). The results of Model 2, adjusted for gender, age and race, showed that serum vitamin D levels were negatively associated with migraine with an OR and 95% CI of 0.74 (0.63, 0.87). Model 3 was adjusted for all covariates. Serum vitamin D in Q4 was significantly associated with migraine, with an OR and 95% CI of 0.84 (0.71, 0.99), respectively. In all three models, p for trend was less than 0.05.

**Table 2:** Relationship between serum vitamin D levels and prevalence of migraine.

	Non-adjusted		Adjust I		Adjust II	
Variable	OR (95%CI)	P value	OR (95%CI)	P value	OR (95%CI)	P value
serum vitamin D(continuous)	1.00 (0.99, 1.00)	< 0.001	0.99 (0.99, 1.00)	0.002	1.00 (0.99, 1.00)	0.01
serum vitamin D(quartile)						
Quartile 1	Ref		Ref		Ref	
Quartile 2	0.90 (0.78, 1.03)	0.14	0.96 (0.83, 1.12)	0.64	1.02 (0.87, 1.19)	0.82
Quartile 3	0.74 (0.64, 0.86)	< 0.001	0.81 (0.69, 0.94)	< 0.001	0.89 (0.76, 1.04)	0.15
Quartile 4	0.77 (0.67, 0.89)	< 0.001	0.74 (0.63, 0.87)	< 0.001	0.84 (0.71, 0.99)	0.04
P for trend	<0.001		<0.001		0.02	

Model 1: No covariates were adjusted. Model 2: Age, gender, and race were adjusted. Model 3: Age, gender, race, Education level, BMI, PIR, Hypertension, Hyperlipidemia, Diabetes, Stroke, Drinking status, and Smoke status were adjusted.

Q, quartile; PIR, ratio of family income to poverty; BMI, body mass index.

### Subgroup analysis

**Fig 2:**
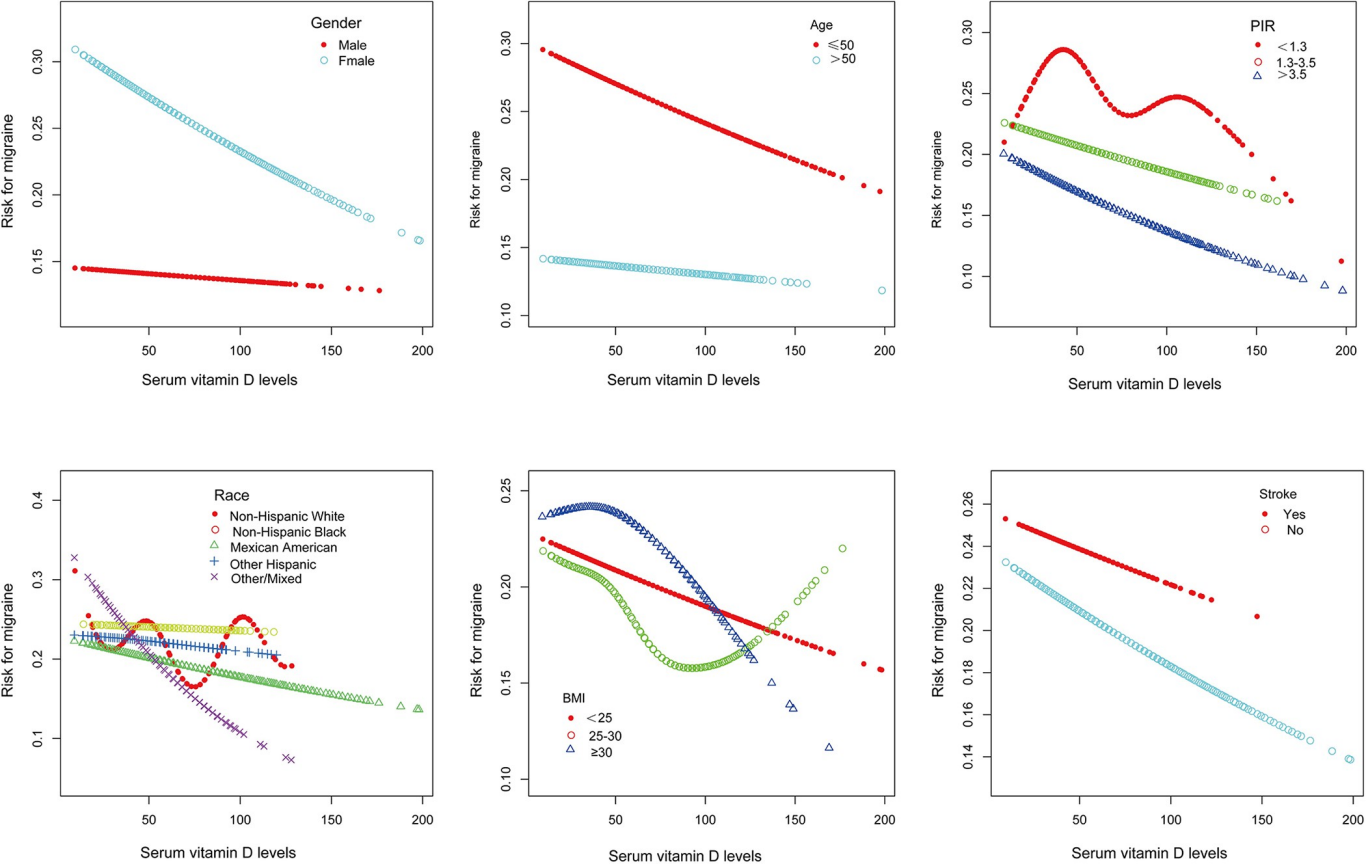
The associations between serum vitamin D and Migraine were stratified by age, gender, race, body mass index, PIR, and stroke.

Subgroups were analyzed by age, gender, race, body mass index, PIR, and stroke. Interaction tests showed that the relationship between serum vitamin D and migraine was not statistically different between strata (interaction test p > 0. 05) as shown in Table 3. Smoothed curves were also fitted according to the subgroups of gender, age, PIR, ethnicity, body mass index, and stroke see Fig 2.

**Table 3:** Subgroup analysis of the association between serum vitamin D and prevalence of migraine.

Subgroup	n	OR (95% CI)	P for interaction
Gender			0.21
Male	4387	1.00 (0.99, 1.00)	
Female	4755	1.00 (0.99, 1.00)*	
Age			0.40
≤50	4940	1.00 (0.99, 1.00)*	
>50	4202	1.00 (0.99, 1.00)	
PIR			0.66
<1.3	2567	1.00 (0.99, 1.00)	
1.3–3.5	3606	1.00 (0.99, 1.00)	
>3.5	2969	1.00 (0.99, 1.00)*	
Race			0.55
Non-Hispanic White	1891	1.00 (0.99, 1.00)	
Non-Hispanic Black	334	1.00 (0.99, 1.01)	
Mexican American	4878	1.00 (0.99, 1.00)	
Other Hispanic	1693	1.00 (0.99, 1.01)	
Other/Mixed	346	0.98 (0.97, 1.00)*	
BMI			0.62
<25	2910	1.00 (0.99, 1.00)	
25–30	3334	1.00 (0.99, 1.00)*	
≥30	2898	1.00 (0.99, 1.00)	
Stroke			0.77
Yes	321	1.00 (0.99, 1.01)	
No	8821	1.00 (0.99, 1.00)*	

In subgroup analyses stratified by gender, age, PIR, Race, BMI, and Stroke. The model adjusted for covariates such as age, gender, race, Education level, BMI, PIR, Hypertension, Hyperlipidemia, Diabetes, Stroke, Drinking status, and Smoking status, but the model did not adjust for the stratification variables themselves.

PIR, the ratio of family income to poverty; BMI, body mass index.

* *p*<0.05; *p* < 0.05 was considered statistically significant.

## Analysis

This study examined the relationship between serum vitamin D and severe headaches or migraine in 9,142 US adults by analyzing cross-sectional data from the NHANES program conducted between 2001 and 2004. Even after controlling for possible confounders such as gender, age, race, the ratio of family income to poverty, education, BMI, hypertension, hyperlipidemia, diabetes, stroke, drinking, and smoking, we discovered that raising blood vitamin D levels was inversely linked with migraine outcome. This result was corroborated by subgroup analysis and smoothed curve fitting.

The following studies are consistent with our findings. By using a case-control study, Celikbile et al. [18] discovered that serum vitamin D levels were significantly lower in migraine patients than in normal participants. In addition, another clinical study found that patients with vitamin D deficiency had a significantly higher incidence of aura, phonophobia/phonophobia, autonomic manifestations, anisocoria, and resistance compared to healthy subjects [14]. A meta-analysis that was released in 2020 revealed that migraineurs have lower blood vitamin D levels than the general population and suggested that migraineurs use vitamin D supplements [19]. Not only that, but Ioannidou et al. [20] demonstrated that vitamin D is also significantly associated with migraine in children by means of a systematic evaluation of the existing literature. Niu et al. [21] similarly demonstrated that elevated vitamin D levels reduce the risk of migraine through two-sample Mendelian randomization.

In contrast, Kjaergaard et al. [22] did not find a significant association between migraine and serum 25(OH)D in a cross-sectional study of included headache populations based on smoking status and controlling for confounders such as age, body mass index (BMI), gender, and season. In addition, a study comparing plasma levels of 25-hydroxyvitamin D [25(OH)D] in migraine patients and controls did not show significant differences, and the results suggest that there is no significant association between migraine and vitamin D [23]. Consistent with these results, Wu et al. [24] showed that patients with widespread chronic pain, muscle pain, and arthritis had lower serum concentrations of vitamin D than controls; however, no differences were found in the sera of headache patients.

Ninety percent of the fat-soluble vitamin D is produced by UV light exposure on the skin [25]. According to studies, the body’s circulation carries the active form of vitamin D throughout, where it binds to the vitamin D receptor (VDR) to control calcium and phosphorus metabolism, increase neuronal cell proliferation and differentiation, and strengthen the immune system [26]. It performs specific functions in the central nervous system by influencing cell proliferation, acting as a neuroprotectant and potent antioxidant, and affecting neurotransmitters [27]. One possible underlying cause of migraines is insufficient amounts of vitamin D.

Migraine pathophysiology remains incompletely understood. At the moment, migraine is thought to be caused by the trigeminal vascular system, which is a significant pathogenic mechanism. Vasoactive peptides like substance P and calcitonin gene-related peptide (CGRP) are released in greater amounts when trigeminal ganglia and fibers are activated. These reactive chemicals have the potential to generate aseptic inflammation and throbbing headaches by vasodilatation [28–31]. Vasodilation, platelet activation, plasma protein leakage, and further release of inflammatory chemicals are all possible effects of neurotransmitters on the vascular wall [32,33]. Furthermore, research has demonstrated a possible correlation between the onset of migraine and lifestyle choices, environmental variables, obesity, and vitamin D deficiency [34,35].

The link between vitamin D and migraine is underpinned by intricate and diverse processes. First, by blocking the production of pro-inflammatory cytokines including interleukin 2 (IL-2), IL-12, interferon-γ, and tumor necrosis factor-alpha (TNF-α) mediated by helper T cells, vitamin D regulates the immune system and the inflammatory response [36,37]. A correlation has been shown between migraine episodes and elevations in these pro-inflammatory markers, specifically about neurovascular inflammation in intracerebral and extracerebral arteries [38,39]. An increased release of interleukin and a pro-inflammatory condition can result from a vitamin D deficiency, which can intensify the inflammatory response associated with migraines. Second, a neurotransmitter called 5-hydroxytryptamine (5-HT) is crucial to the etiology of migraines [40]. Tryptophan hydroxylase (TPH), a synthetic enzyme of 5-HT, is modulated by vitamin D, which can impact 5-HT levels [41,42]. Research indicates that vitamin D increases the production of 5-HT in the brain by activating the VDR of TPH2 in the brain and inhibiting the VDR of TPH1 in peripheral tissues [43]. Lastly, by controlling vascular function, endothelial cells have a role in the pathophysiology of migraine. Nitric oxide (NO) production and release are two functions of endothelial cells that are influenced by vitamin D. The pathophysiology of migraines is significantly influenced by NO, and decreased NO production may be linked to compromised endothelial function [44]. The course and severity of migraine episodes may be indirectly influenced by vitamin D due to its effects on endothelial cell metabolism and function [45].

There are several advantages to our study. We made use of a sizable sample of nationally representative NHANES data. Furthermore, we conducted multivariate logistic regression analyses, controlling for pertinent factors, to investigate the separate impacts of blood vitamin D levels on migraine. We also carried out a subgroup analysis. The following limitations exist in this study:(1) Due to the cross-sectional design of this study, causality could not be determined. (2) The study data relied on self-reports from the NHANES Pain Questionnaire, which may have led to recall bias or misclassification. (3) Although this study focused on vitamin D, other nutrients may also have an impact on migraine, so future studies should consider analyzing the interactions between different micronutrients in depth.

Future studies should focus on exploring the causal relationship between vitamin D and migraine, especially considering individuals’ baseline vitamin D levels and migraine severity. Long-term randomized controlled trials are recommended to assess the efficacy of vitamin D supplementation as an intervention for migraine symptoms. Meanwhile, in-depth analyses of vitamin D metabolic pathways and how genetic factors influence its relationship with migraine will help to reveal underlying mechanisms and provide personalized preventive or therapeutic strategies.

## Conclusion

Our study showed a significant negative correlation between serum vitamin D levels and the prevalence of migraine in American adults.

## Supporting information

S1 Data (XLS)
